# Prevalence and clinical characteristics of hospitalized children with community-acquired *Mycoplasma pneumoniae* pneumonia during 2017/2018, Chengde, China

**DOI:** 10.1097/MD.0000000000023786

**Published:** 2021-02-05

**Authors:** Meng Su, Qian Wang, Dan Li, Ling-Ling Wang, Chun-Yang Wang, Jiang-Li Wang, Qing Zhang, Luan-Ying Du, Jian-Ying Liu, Guang-Cheng Xie

**Affiliations:** a Department of Pathogenic Biology; b Department of Respiratory, Children′s Hospital of Hebei Province, Shijiazhuang; c Department of Preventive Medicine, Chengde Medical University; d Clinical Medical College, Xi’an Medical University, Xi’an; e Chengde Center for Disease Control and Prevention, Department of Microbiology Laboratory; f Chinese Center for Viral Disease Control and Prevention, National Institute for Viral Disease Control and Prevention, Beijing; g Department of Pediatrics, NO.2 Clinical Teaching Hospital Affiliated to Chengde Medical University, Chengde, China.

**Keywords:** clinical characteristics, coinfection, community acquired-pneumonia, hospitalized children, *Mycoplasma pneumoniae*

## Abstract

Community acquired-pneumonia (CAP) has varying causative pathogens and clinical characteristics. This study investigated the prevalence of *Mycoplasma pneumoniae* (*M pneumoniae*) and evaluated the clinical characteristics in infected hospitalized children by disease severity.

From throat swabs of hospitalized children (5 months to 14 years) with CAP collected between November 2017 and May 2018, *M pneumoniae* and other CAP pathogens were identified using polymerase chain reaction (PCR). Differences in clinical and laboratory test data were compared between severe and mild case groups.

Of 333 hospitalized children enrolled, 221/333 (66.4%) tested positive for *M pneumoniae* and 24/221 (10.9%) patients were (n = 9, aged <5 years vs n = 15, ≥5 years) single infection by PCR, however, only 170/333 (51.1%) patients were presented with *M pneumoniae* IgM-positive. *M pneumoniae* detection rate by PCR was higher than by immunoglobulin (IgM) serology. In 123/221 (55.7%) *M pneumoniae* infected patients, coinfection with bacterial pathogens (n = 61, <5 years vs n = 62, ≥5 years) occurred. Children (aged 3–8 years) had most *M pneumoniae* infection. Severe *M pneumoniae* pneumonia (MPP) in children occurred mostly in older age (7 [interquartile ranges {IQR}, 6–8] years; *P* < .0001), with longer cough days (14 [IQR, 10–19.5] days; *P* = .002) and hospitalization duration (9.5 [IQR, 7–12.3] days; *P* < .0001), lower lymphocyte ratio (24.1, [IQR, 20.0–31.1] %; *P* = .001), higher neutrophils ratio (66.0, [IQR, 60.2–70.3]%; *P* < .0001), and serum C-reactive protein (CRP) level (3.8, [IQR, 1.3–10.9] mg/L; *P* = .027).

*M pneumoniae* is the most commonly detected pathogen in CAP. High coinfection prevalence increases diagnosis difficulty by clinically nonspecific characteristics. *M pneumoniae* detection by PCR with IgM may improve precise and reliable diagnosis of community-acquired MPP.

## 1 Introduction

Community-acquired pneumonia (CAP), a leading infectious disease, is caused by multiple pathogens.^[1]^ Bacteria usually cause “typical pneumonia,” while “atypical pathogens” including *Mycoplasma pneumoniae* (*M pneumoniae*), *Chlamydophilia pneumoniae* (*C pneumoniae*), and *Legionella pneumophila* (*L pneumophila*) cause “atypical pneumonia.” These 3 pathogens account for 21% to 28% of adult CAP worldwide.^[2,3]^

*M pneumoniae* is a major pathogen causing respiratory tract infection and atypical pneumonia at any age.^[4]^*M pneumoniae* infections remain one of the most common causes of CAP, but the prevalence of *M pneumoniae* is usually underestimated because in patients with pneumonia caused by *M pneumoniae* is usually asymptomatic. Hence, *M pneumoniae* pneumonia (MPP) is considered to be the “walking pneumonia.”^[4]^ A prospective study of pathogens associated with pneumonia in adults (aged ≥19 years) in 7 cities (Beijing, Shanghai, Shenyang, Xi’an, Chengdu, Guangzhou, and Hangzhou) in China indicated *M pneumoniae* is the major causative agent of CAP with a prevalence rate of up to 20.7%.^[5,6]^ Prevalence of *M pneumoniae* in hospitalized children with acute respiratory infection showed *M pneumoniae* to be the dominant pathogen with the highest detection rate (56.9%) among 10,435 specimens in Hubei, between May 2010 and April 2012.^[7]^ Another study also indicated *M pneumoniae* as the most predominant pathogen with a positive rate of 40.78% among 1204 children aged 4 to 14 years, between August 2011 and August 2013 in Nanjing, China.^[8]^

MPP usually presents with fever, cough, diarrhea, and other non-specific symptoms,^[9,10]^ however, MPP remains largely underdiagnosed. Lack of diagnostic test methods and other infections which either coexist or mimic *M pneumoniae* limit the understanding of the burden and epidemiology of hospitalized MPP. Case-control data indicate that 93.0% of cases and 74.1% of controls have >1 detected microorganisms in children <5 years with pneumonia.^[11]^ Thus, determination of the etiological profile of MPP and the relationship with clinical features may provide information for improving the management and treatment of MPP.

This study aimed to determine the prevalence of *M pneumoniae* in hospitalized children with CAP at the No. 2 Clinical Teaching Hospital affiliated to Chengde Medical University and to evaluate differences in clinical characteristics of hospitalized children with MPP between severe and mild case groups.

## 2 Materials and methods

### 2.1 Study patients

A total of 333 hospitalized children between 5 months and 14 years who were admitted to the No.2 Clinical Teaching Hospital (affiliated to Chengde Medical University [Chengde, China]) with CAP were enrolled from November 2017 to May 2018.

### 2.2 Samples and clinical data collection

Throat swabs were collected from enrolled hospitalized children with CAP and stored in 2 mL germ free physiological saline. A total of 333 throat swabs were collected. The fresh samples were stored at –25 °C. Medical records of enrolled hospitalized children were reviewed and general clinical data including admission number, month of admission, sex, age, distribution of residence, use of antibiotics were also collected. Additional clinical data associated with disease severity such as: peak of fever, duration of fever, and duration of hospitalization were also collected. The laboratory data of white blood cell (WBC) count, neutrophils count, lymphocytes count, concentration of C-reactive protein (CRP), concentration of lactate dehydrogenase (LDH) were collected. The enrolled patients were divided into 2 groups: based on age (<5 years and ≥5 years), and disease severity (severe case group and mild case group), and the clinical data were compared. Written informed consent was obtained from the guardians of all study subjects and the study design was approved by Chengde Medical University (No.2017020).

### 2.3 Radiological assessment

Chest radiographs (chest x-ray or computed tomography scan) of the hospitalized children were done upon admission and the results were interpreted by 2 professional radiologists, and the final assessments were made by consensus of 2 pediatricians in accordance with the standardized definition of radiological pneumonia.^[12]^

### 2.4 Nucleotide extraction

DNA was extracted from 300 μL of throat swabs using QIAamp DNA mini kit (QIAGEN, Hilden, Germany). Viral DNA and RNA were extracted from 200 μL of throat swabs using Viral Nucleic Acid Extraction Kit II (Geneaid, New Taipei, China Taiwan). All processes were done according to the manufacturer's instructions. The nucleic acids were stored at –40 °C until further detection of bacterial or viral pathogens.

### 2.5 Detection of *M pneumoniae* by PCR

The 16S rDNA was amplified as the target gene of *M pneumoniae* by PCR amplification. The Mpn primer 1 (AAGGACCTGCAAGGGTTCGT) and Mpn primer 2 (CTCTAGCCATTACCTGCTAA) were as previously described.^[13]^ The PCR amplification was conducted using 2×Easy-Load PCR Master Mix (Beyotime, Shanghai, China) in a final volume of 20 μL with 5 μL template DNA to generate 277 bp amplification fragment. The condition of PCR amplification was as follows: initial denaturation at 95 °C for 4 minutes; 40 cycles of denaturation 1 minute at 95 °C, annealing 1 minute at 60 °C, and extension 1.5 minutes at 72 °C, finally extension 10 minutes at 72 °C. The PCR products were screened using agarose gel electrophoresis with NA-Red staining (Beyotime, Shanghai, China) and the positive PCR products were sequenced by Tianyihuiyuan Co., Ltd. (Beijing, China).

### 2.6 Diagnostic for other respiratory pathogens

Bacterial pathogens including *C pneumoniae*,^[14]^*L pneumophila*,^[15]^ group A Streptococcus (GAS),^[16]^*Klebsiella pneumoniae* (*K pneumoniae*),^[17]^*Staphylococcus aureus* (*S aureus*),^[18]^*Pseudomonas aeruginosa* (*P aeruginosa*),^[19]^*Haemophilus influenzae* (*H influenzae*)^[20]^ were detected by PCR or real-time PCR. Additionally, viral pathogens including influenza A/B/C viruses^[21]^; parainfluenza virus (PIV) 1, 2, and 3^[22]^; adenovirus (AdV)^[23]^; human bocavirus (HBoV)^[24]^; human rhinovirus (HRV)^[25]^; human metapneumovirus (hMPV)^[26]^; respiratory syncytial virus (RSV)^[21]^; human coronavirus (HCoV)^[27]^ were also detected by polymerase chain reaction or reverse transcription-polymerase chain reaction. The primers are described in the reference articles.

### 2.7 Immunoglobulin determination for *M pneumoniae*

The presences of *M pneumoniae* immunoglobulin (IgM) in peripheral blood of hospitalized children with CAP were tested by using the IgM-specific detection kit (VIRCELL, Granada, Spain). Nine pathogens (*L pneumophila*, *M pneumoniae*, Q-fever Rickettsia burneti, *C pneumoniae*, AdV, RSV, influenza A viruses [Flu A], influenza B viruses [Flu B], PIV 1-3) were tested for by indirect immunofluorescence assays (IFA). Tests and interpretation of results were in accordance with the manufacturer's instructions.

### 2.8 Case definitions and enrollment criteria

Patients having the following presentations were defined as having CAP, such as fever and body temperature >37.8 °C, cough, rales, or the presence of consolidation, infiltrate and pleural effusion supported by radiographic evidence. Hospitalized children with positive for *M pneumoniae* IgM or nucleic acid by PCR were defined as MPP. Patients who met one of the following criteria were defined as severe MPP, such as necrotizing pneumonia, dysfunction in respiratory tract or complication in other systems, complicated bronchiolitis obliterans, complicated systemic inflammatory response syndrome, less therapeutic response to single macrolides, persistent cough, and lung rale >1 week. Patients were excluded from this study if the age at hospitalization was ≤28 days, or ≥15 years; diagnosed with malignant tumor, or other pulmonary illness; or patients’ history was missing or incomplete.

*M pneumoniae* CAP was confirmed if the sample was positive for *M pneumoniae* PCR or IgM. With negative *M pneumoniae* PCR or IgM, the patient was considered to have CAP without *M pneumoniae*. With positive PCR detection of bacterial or viral pathogens, the patient was considered to have bacterial or viral CAP. Coinfection of *M pneumoniae* CAP was defined as the patient with ≥1 other bacterial or viral CAP-associated pathogen.

### 2.9 Statistical analysis

The database was generated in Microsoft Excel 2010 (Microsoft Corporation, CA). The categorical variables were presented as counts and percentages, and the continuous variables were described as median and interquartile ranges (IQR). The chi-squared test or Fisher exact test was used in comparisons of categorical variables. The Mann–Whitney *U* test was used to compare the difference of continuous variables. The significant difference was considered as the level of *P *< .05 with 2-tailed test. All statistical analyses were performed with the IBM SPSS Statistics 25.0 software package (IBM corp., Armonk, NY).

## 3 Results

### 3.1 Prevalence of *M pneumoniae*

**Figure 1:**
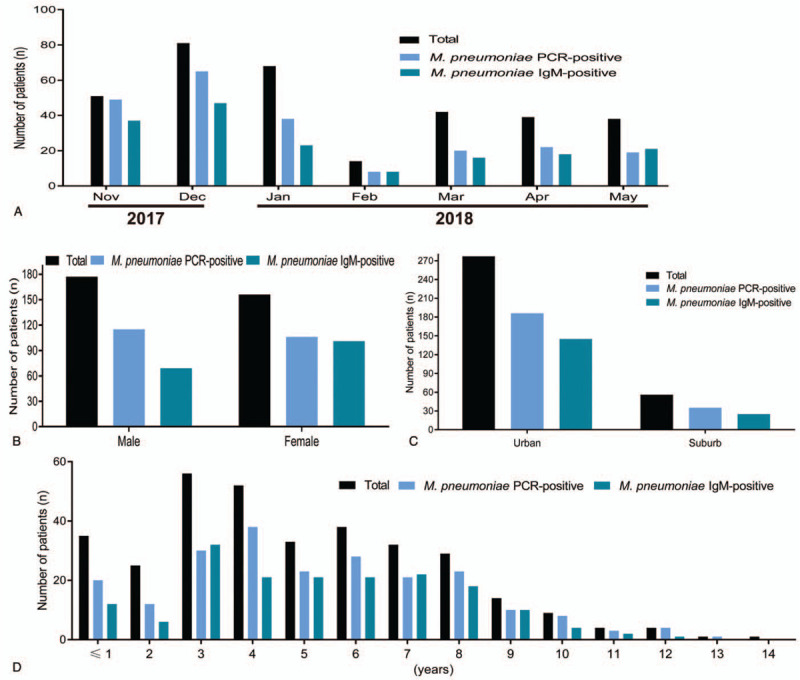
Prevalence of *M pneumoniae* in hospitalized children with CAP detected by PCR and IgM test. (A) Monthly distribution of *M pneumoniae* during 2017 to 2018; (B) gender distribution of *M pneumoniae*; (C) residence distribution of *M pneumoniae*; (D) age distribution of *M pneumoniae*. CAP = community acquired-pneumonia.

Among the 333 hospitalized children with CAP, a total of 221/333 (66.4%) patients were positive for *M pneumoniae* genomic DNA by PCR detection, however, only 170/333 (51.1%) patients were presented with *M pneumoniae* IgM. The monthly distribution of *M pneumoniae* occurred throughout the study period from November 2017 to May 2018 and was peaked at December 2017 (Fig. 1A). A total of 115/221 (52.0%) male patients were infected with *M pneumoniae* detected by PCR, but there only 69/170 (40.6%) male patients were *M pneumoniae* IgM-positive. A total of 106/221 (48.0%) and 101/170 (59.4%) female patients were *M pneumoniae* PCR-positive and *M pneumoniae* IgM-positive, respectively (Fig. 1B). The residence distribution of patients showed that 186/221 (84.2%) and 145/170 (85.3%) patients infected with *M pneumoniae* detected by PCR and IgM were distributed in urban region of Chengde (Fig. 1C). Patients (age ranged 5 months to 13 years) were infected with *M pneumoniae* with most being 3 to 8 years of age, especially hospitalized children aged 3 to 4 years of age (Fig. 1D).

A total of 125/333 (37.5%) patients were positive with *M pneumoniae* both detection of PCR and IgM, 45/333 (13.5%) patients were positive for IgM of *M pneumoniae* but were PCR negative, however, 96/333 (28.8%) patients were positive for *M pneumoniae* with detection of PCR but were IgM negative, and 67/333 (20.1%) patients were both negative for *M pneumoniae* both detection of PCR and IgM (Table 1).

**Table 1:** Sensitivity comparison between PCR and serology in detection of *M pneumoniae*.

Method	PCR positive	PCR negative	Total
Serology positive, n (%)	125 (37.5)	45 (13.5)	170 (51.1)
Serology negative, n (%)	96 (28.8)	67 (20.1)	163 (48.9)
Total	221 (66.4)	112 (33.6)	333 (100)

### 3.2 Epidemiology of *M pneumoniae* infection

The seasonal distribution of hospitalized children with MPP indicated that 160/221 (72.4%) patients were infected with *M pneumoniae* from November 2017 to February 2018; however, 61/221 (27.6%) patients were infected with *M pneumoniae* from March 2018 to May 2018 (Table 2). These results indicated that the prevalence rate of *M pneumoniae* in hospitalized children was higher in winter than spring. The patients were divided into 2 groups, 100/221 (45.2%) patients infected with *M pneumoniae* were <5 years, and 121/221 (54.8%) patients infected with *M pneumoniae* were ≥5 years (Table 3).

**Table 2:** Seasonal distribution of *M pneumoniae* in hospitalized children with MPP.

Oneset season	Total	Positive	Negative
Nov–Feb, n (%)	214	160 (72.4)	54 (48.2)
Mar–May, n (%)	119	61 (27.6)	58 (51.8)
Total	333	221	112

**Table 3:** Age distribution of *M pneumoniae* in hospitalized children with MPP.

Age group	Total	Positive	Negative
<5 years, n (%)	168	100 (45.2)	68 (60.7)
≥5 years, n (%)	165	121 (54.8)	44 (39.3)
Total	333	221	112

### 3.3 Coinfections

A total of 24/221 (10.9%) patients were single infection among 221 patients positive for *M pneumoniae* infection (*P* = .419). Mixed infections (including *C pneumoniae*, viral and bacterial coinfections) were identified in 197/221 (89.1%) of studied cases. Bacterial and viral coinfection were found in 123/221 (55.7%) and 12/221 (5.4%) of cases, respectively. Viral/bacterial coinfection with *M pneumoniae* were identified in 59/221 (26.7%) cases (Table 4).

**Table 4:** Age distribution of bacterial and viral pathogens coinfected with *M pneumoniae*.

	<5 yearsn = 100n (%)	≥5 yearsn = 121n (%)	Totaln = 221n (%)	*P*	OR (95%CI)
*M pneumoniae*	9 (9.0)	15 (12.4)	24 (10.9)	.419	0.699 (0.292–1.673)
*M pneumoniae* + *C pneumoniae*	0 (0)	3 (2.5)	3 (1.4)	.253	–
*M pneumoniae* + bacteria^∗^	61 (61.0)	62 (51.2)	123 (55.7)	.146	1.488 (0.870–2.547)
*M pneumoniae* + virus^¶^	5 (5.0)	7 (5.8)	12 (5.4)	.798	0.857 (0.264–2.788)
*M pneumoniae* + bacteria + virus	25 (25.0)	34 (28.1)	59 (26.7)	.604	0.853 (0.467–1.557)

### 3.4 Clinical characteristics

Severe cases were older than mild cases (median, 7 [IQR, 6–8] years vs 4 [IQR, 3–7] years; *P* < .0001). The severe cases were present with higher peak of fever (39.6, [IQR, 39.1–40.0] °C; *P* < .0001); longer duration of fever (8, [IQR, 5–10] days; *P* < .0001), cough (15, [IQR, 12–21] days; *P* < .0001), and hospitalization (11, [IQR, 9–14] days; *P* < .0001) compared with mild cases. The severe cases with lower WBC count (7.4, [IQR, 6.4–8.8] × 10^9^ cells/L; *P* = .003) and absolute lymphocyte count (23.8, [IQR, 18.1–29.7]%; *P* < .0001), but with higher absolute neutrophil count (66.0, [IQR, 60.7–70.6]%; *P* < .0001) and serum CRP level (3.1, [IQR, 1.3–12.0] mg/L; *P* < .0001) (Table 5).

**Table 5:** Clinical characteristics of hospitalized children with CAP between severe and mild case groups.

	Severe cases(n = 40)	Mild cases(n = 293)	*P*
Demographic and clinical presentation			
Males, n (%)	18 (45.0)	159 (54.3)	.271
Age (yrs), median (IQR)	7 (6–8)	4 (3–7)	<.0001
Peak of fever (°C), median (IQR)	39.6 (39.1–40.0)	39.2 (38.8–39.7)	<.0001
Duration of fever (days), median (IQR)	8 (5–10)	5 (3.5–8)	<.0001
Cough (days), median (IQR)	15 (12–21)	11 (9–14)	<.0001
Rales, n (%)	18 (45.0)	135 (46.1)	.898
Clinical outcome			
Duration of hospitalization (days), median (IQR)	11 (9–14)	6 (5–8)	<.0001
Laboratory data			
WBC (×10^9^ cells/L), median (IQR)	7.4 (6.4–8.8)	7.7 (5.9–10.7)	.003
Neutrophils (%), median (IQR)	66.0 (60.7–70.6)	58.2 (47.0–67.2)	<.0001
Lymphocytes (%), median (IQR)	23.8 (18.1–29.7)	30.0 (21.8–40.9)	<.0001
CRP (mg/L), median (IQR)	3.1 (1.3–12.0)	2.2 (0.6–8.7)	<.0001
LDH (U/L), median (IQR)	252 (226–312)	263 (227–318)	.330

Severe MPP cases were older than the mild MPP cases (7 [IQR, 6–8] years vs 4 [IQR, 3–7] years; *P* < .0001). Duration of fever (7 [IQR, 4.8–10] days; *P* = .011), cough (14 [IQR, 10–19.5] days; *P* = .002), and duration of hospitalization (9.5 [IQR, 7–12.3] days; *P* < .0001) were longer in severe MPP cases. The severe MPP cases with lower ratio of lymphocytes (24.1 [IQR, 20.0–31.1] %; *P* = .001), but with higher ratio of neutrophils (66.0, [IQR, 60.2–70.3]%; *P* < .0001) and serum CRP level (3.8, [IQR, 1.3–10.9] mg/L; *P* = .027) (Table 6).

**Table 6:** Clinical characteristics of hospitalized children with MPP between severe and mild case groups.

	Severe casesn = 33	Mild casesn = 188	*P*
Demographic and clinical presentation			
Males, n (%)	16 (48.5)	99 (52.7)	.658
Age (years), median (IQR)	7 (6–8)	4 (3–7)	<.0001
Peak of fever (°C), median (IQR)	39.5 (39.0–40.0)	39.2 (38.8–39.6)	.061
Duration of fever (days), median (IQR)	7 (4.8–10)	5 (3–7)	.011
Cough (days), median (IQR)	14 (10–19.5)	10 (8–13)	.002
Rales, n (%)	16 (48.5)	99 (52.7)	.658
Clinical outcome			
Duration of hospitalization (days), median (IQR)	9.5 (7–12.3)	6 (5–7)	<.0001
Laboratory data			
WBC (×10^9^cells/L), median (IQR)	7.7 (6.5–9.0)	7.3 (5.6–10.7)	.717
Neutrophils (%), median (IQR)	66.0 (60.2–70.3)	57.4 (46.5–66.9)	<.0001
Lymphocytes (%), median (IQR)	24.1 (20.0–31.1)	30.5 (23.1–41.1)	.001
CRP (mg/L), median (IQR)	3.8 (1.3–10.9)	1.7 (0.5–6.8)	.027
LDH (U/L), median (IQR)	251 (219–306)	261 (226–313)	.672

## 4 Discussion

In this study, we investigated the prevalence and clinical characteristics of hospitalized children infected with *M pneumoniae.* Our results showed that *M pneumoniae* was the dominant pathogen with different clinical characteristics of hospitalized children with MPP between severe and mild case groups, such as age, duration of fever, cough, duration of hospitalization, neutrophils, lymphocytes, and serum CRP level.

The etiology studies of acute respiratory tract infections (ARTI) in Hubei province showed that *M pneumoniae* were the leading causative agent in children with ARTI at Renmin Hospital of Wuhan University between May 2010 and April 2012,^[7]^ and at Wuhan Children's Hospital between October 2010 and September 2012.^[28]^ Children with ARTI in Guangzhou Women and Children's Medical center showed that *M pneumoniae* (33.15%) was also the top pathogen between 2011 and 2012.^[29]^*M pneumoniae* (40.78%) was the dominant pathogen in children with CAP at Zhongda Hospital in Nanjing between August 2011 and August 2013.^[8]^*M pneumoniae* (38.47%) was also the dominant pathogen at the affiliated Hospital of Southwest Medical University in Luzhou from July 2013 to December 2016.^[30]^ In our study, 221 children were positive for *M pneumoniae.* Meanwhile, *H influenzae* and GAS were (209 children were positive for *H influenzae* and 125 children were positive for GAS) the 2 leading bacterial pathogens. hMPV and HBoV (80 cases were infected with hMPV, and 22 cases were infected with HBoV) were the 2 dominant viral pathogens.^[31]^ Our study indicated that *M pneumoniae* is the dominant pathogen of hospitalized children with CAP in Chengde, China between 2017 and 2018. The key finding of this study is the increasing prevalence of *M pneumoniae* in hospitalized children with CAP.

*M pneumoniae* is usually associated with infections in school-aged children and young adults,^[32]^ however, the epidemiology and disease burden of hospitalized children with CAP caused by *M pneumoniae* is poorly understood. Currently, *M pneumoniae* is detected mainly by using the presence of IgM at admission or a ≥4-fold rise of IgG in serum.^[10]^ Our results showed that 170 hospitalized children were positive for *M pneumoniae* IgM, however, 221 hospitalized children were *M pneumoniae* PCR-positive. *M pneumoniae* could be detected in asymptomatic children with a percentage of 21.2%,^[33]^*M pneumoniae* (0.6%) was also detected in asymptomatic control samples by PCR from hospitalized patients with radiographically confirmed pneumonia.^[34]^ The studies on the prevalence of *M pneumoniae* also used the upper respiratory tract pathogens panel detection kit to test the presence of IgM against other pathogens, this method had confirmed that *M pneumoniae* was the dominant pathogen in children with respiratory tract infections in the cities of mainland China, such as Wuhan,^[7,28]^ Nanjing,^[8]^ and Luzhou.^[30]^ We found the positive rate of *M pneumoniae* detected by PCR was higher than IgM. Thus, our research indicated that PCR should be the gold standard for diagnosis of *M pneumoniae* although its usage is not widespread in hospitals in mainland China yet. We also support the opinion proposed by previous report^[34]^ that *M pneumoniae* is likely as the causative pathogen in hospitalized children with pneumonia when detected by PCR.

Our results showed that multiple pathogens (5 viruses and 4 bacteria) were coinfected with *M pneumoniae* among the hospitalized children with CAP. *M pneumoniae* coinfected with viral pathogens (RSV, AdV, PIV, and FluB)^[7,10,35]^ or bacterial pathogen (*S pneumoniae*)^[36]^ were also detected. At hence, we conclude that double and triple infections with *M pneumoniae* are common events.^[11]^

Previous study showed that children aged 2 to 5-year-old were more susceptible to *M pneumoniae*,^[30]^ other study also confirmed that children >1-year-old were more prone to *M pneumoniae*, especially children aged 3 to 6-year-old.^[7]^ Our results showed that children aged ≥5 years had higher detection rate of *M pneumoniae* than children <5 years, but children aged 3 to 8 years were the major target for *M pneumoniae*. The monthly distribution of *M pneumoniae* showed that October 2010 and October 2011 to December 2011 were the epidemic peak period,^[32]^ however, another study showed that summer (June) and autumn (September) had high positive percentage of *M pneumoniae.*^[8]^ One study showed the prevalence of *M pneumoniae* was relatively increased from November to January annually and peaked in winter (November 2013, December 2014 and 2015).^[30]^ Our results showed that monthly distribution of *M pneumoniae* was observed throughout November 2017 and May 2018. Our result also confirmed that winter was high prevalence time of *M pneumoniae* and that it peaked in December 2017.

In this study, we compared the clinical features of MPP children between severe and mild case groups, we found the severe MPP cases had longer duration of hospitalization (9.5 days), higher neutrophils (66.0%), lower lymphocytes (24.1%), and higher serum CRP level (3.8 mg/L) compared with mild MPP cases. Clinical characteristics of children infected with *M pneumoniae* indicated that the children usually had more frequent cough, headache, and wheezing compared with non-*M pneumoniae* infected children, but the WBC, platelet, and CRP levels had no significant difference between the study groups.^[37]^ Other study indicated *M pneumoniae* PCR-positive children usually presented with the following clinical presentation, such as fever, fatigue, chills, headache, sore throat, wheezing, runny nose, abnormal pain, chest pain. Examination findings included decreased breath sounds, rales, chest indrawing, rhonchi, and wheezing, however, laboratory test such as abnormal WBC count, abnormal platelet count, and hyponatremia had no significant difference between the study groups.^[34]^ So, the clinicians could not distinguish *M pneumoniae* from viral or bacterial pneumonia just judging by clinical symptoms, signs, and the radiographic tests. We recommend using serology test combined with PCR assay for diagnosing hospitalized children with pneumonia, this will improve the quality of treatment and decrease the disease burden of patients.

This study was limited by the small number of samples (total 333 samples were collected from November 2017 to May 2018), which may have resulted in some loss in prevalence of *M pneumoniae* in hospitalized children throughout the year. Additionally, further studies such as using P1 gene or multilocus variable-number tandem repeat analysis (MLVA) to determine the genotypes of *M pneumoniae*, and determining antibiotic resistance especially macrolide-resistant *M pneumoniae* or minimum inhibitory concentrations of macrolides, are needed to conduct. Although this study had some above limitations, these limitations also point out our works’ focuses in the next step.

## 5 Conclusion

In conclusion, our study shows that *M pneumoniae* is the dominant pathogen of hospitalized children with CAP and winter is the peak prevalence time. We recommend that the use of PCR detection method may further improve etiological diagnosis of *M pneumoniae* where IgM detection is currently used.

## Acknowledgments

The authors thank all enrolled hospitalized children and guardians in this study. Also, we thank all doctors and nurses who have participated in this study.

## Author contributions

**Conceptualization:** Guang-Cheng Xie, Jian-Ying Liu, Luan-Ying Du.

**Data curation:** Meng Su, Qian Wang, Ling-Ling Wang, Dan Li.

**Formal analysis:** Meng Su, Qian Wang, Ling-Ling Wang, Dan Li, Chun-Yang Wang.

**Investigation:** Meng Su, Qian Wang, Ling-Ling Wang, Dan Li.

**Methodology:** Meng Su, Qian Wang, Ling-Ling Wang, Dan Li, Jiang-Li Wang, Qing Zhang.

**Project administration:** Guang-Cheng Xie, Chun-Yang Wang.

**Supervision:** Guang-Cheng Xie, Luan-Ying Du.

**Validation:** Jiang-Li Wang, Qing Zhang.

**Writing – original draft:** Meng Su, Qian Wang.

**Writing – review & editing:** Guang-Cheng Xie, Jian-Ying Liu, Chun-Yang Wang.
